# Epidemiological survey in single-species flocks from Poland reveals expanded genetic and antigenic diversity of small ruminant lentiviruses

**DOI:** 10.1371/journal.pone.0193892

**Published:** 2018-03-05

**Authors:** Monika Olech, Stephen Valas, Jacek Kuźmak

**Affiliations:** 1 Department of Biochemistry, National Veterinary Research Institute, Puławy, Poland; 2 Anses Niort Laboratory, Niort Cedex, France; CEA, FRANCE

## Abstract

Small ruminant lentivirus (SRLV) infections are widespread in Poland and circulation of subtypes A1, A12, A13, B1 and B2 was detected. The present work aimed at extending previous study based on the analysis of a larger number of animals from single-species flocks. Animals were selected for genetic analysis based on serological reactivity towards a range of recombinant antigens derived from Gag and Env viral proteins. Phylogenetic analysis revealed the existence of subtypes B2 and A12 in both goats and sheep and subtypes A1 and B1 in goats only. In addition, two novel subtypes, A16 and A17, were found in goats. Co-infections with strains belonging to different subtypes within A and B groups were detected in 1 sheep and 4 goats originating from four flocks. Although the reactivity of serum samples towards the recombinant antigens confirmed immunological relatedness between Gag epitopes of different subtypes and the cross-reactive nature of Gag antibodies, eleven serum samples failed to react with antigens representing all subtypes detected up-to-date in Poland, highlighting the limitations of the serological diagnosis. These data showed the complex nature of SRLV subtypes circulating in sheep and goats in Poland and the need for improving SRLV-related diagnostic capacity.

## Introduction

Caprine arthritis encephalitis virus (CAEV) and Maedi Visna virus (MVV) are small ruminant lentiviruses (SRLV) infecting goats and sheep respectively. In the course of infection the majority of animals remains clinically healthy, however, a fraction of them develops clinical manifestations. While infection leads to mastitis and painful enlargement of the carpal joints (arthritic form) in adult goats and encephalitis in goatlings [1], respiratory disorders such as dyspnea, as well as progressive weight loss and indurative mastitis are more common in sheep [2].

Similarly to other complex retroviruses, the genome of SRLV contains the *gag*, *pol* and *env* genes and additional open reading frames encoding regulatory proteins (*vpr-like*, *vif* and *rev*). The provirus genome is flanked by non-coding long terminal repeat regions (LTRs) composed of U3-R-U5 [3]. For years, CAEV and MVV have been considered to be goat- and sheep-specific pathogens, respectively. However, phylogenetic studies and discoveries of cross-species infections demonstrated that these viruses should be more correctly referred to as SRLV. Classification based on long (>1kb) *gag* and/or *pol* sequences divided these viruses into five groups A to E, of which groups A, B and E contain several different subtypes [4, 5]. Groups A and B are widely distributed throughout the world and referred to as MVV-like and CAEV-like viruses, respectively. Within group A fifteen subtypes (A1–A15) have been recognized so far, whereas group B was divided into four subtypes, B1–B4 [6, 7]. However, a recently published study revealed that strains representing subtype B4 are in fact recombinants named CRF01_AB SRLV [8]. Groups C–E are geographically restricted. Group C comprises viral strains isolated from Norwegian sheep and goats [9] while group D has only been described in Swiss and Spanish sheep based on *pol* sequences [5]. Phylogenetic analysis of *gag* sequences of the same isolates classified these sequences within group A, suggesting that genotype D is in fact genotype A, exhibiting divergence in the *pol* gene [6]. Group E, relatively divergent from any known genotype, has only been detected in the north of Italy and Sardinia and contains two subtypes (E1 and E2) [4, 10].

SRLV infections are commonly diagnosed based on serological tests detecting antibodies against epitopes located in structural proteins. In spite of antigenic variability, cross-reacting antibodies between CAEV and MVV-like antigens have been described [11]. However, several studies demonstrated that two gag-encoded products, capsid (CA) and matrix (MA) proteins, carry group-specific epitopes and that most sheep and goat sera reacted to these antigens in a group-specific manner [12, 13]. Env-encoded surface glycoproteins (SU1 and SU5 immunodominant epitopes) have also been used in serological diagnosis and, due to the high variability of SRLV, these peptides are useful for type-specific diagnosis [14, 15]. The application of a mixture of Gag and Env-derived antigens representing SRLV groups A and B increased the sensitivity of ELISA in diagnosis of SRLV infection [16, 17]. However, mixed infections with isolates from different groups as well as the occurrence of infections with new genetic variants generated by mutations or recombinations may influence serological diagnosis. Therefore, continuous analysis of SRLV genotypes circulating in different flocks is necessary not only for epidemiological analyses but also for evaluation of the potential effect of genetic variability on serological detection.

In a previous study [18]we showed that SRLV strains found in mixed flocks in Poland belonged to the well-known subtypes B1, B2 and A1 as well as to the more recently established subtypes A12 and A13. We also revealed the circulation of more than one subtype in particular flocks and the presence of animals co-infected with viruses belonging to group A and B.

In the present study, we aimed at extending the previous investigation by analysis of the SRLV diversity in a larger number of animals from single-species flocks. In order to identify animals infected with new subtypes or co-infections, the serological reactivity of positive samples towards a range of recombinant antigens representing different SRLV subtypes was characterized. Samples from selected animals were subjected to genetic analysis of both *gag* and *env* genes.

## Materials and methods

### Animals and samples

A total of 20 sheep and 39 goats originating from two and four flocks respectively were investigated in this study. All animals were clinically healthy and came from flocks which were located in four voivodships of Central, East and South of Poland. These flocks were epidemiologically unrelated since no trade and movement of animals were noted between them. All flocks included single animal species with breeds typical for dairy flocks in Poland. Presence of SRLV infection in these flocks was confirmed on the basis of previous serological survey [15, 19]. Blood was taken by jugular venipuncture and collected in EDTA and serum tubes for further analysis. All animal procedures received approval of Local Ethical Committee on Animal Testing at the University of Life Sciences in Lublin (Poland). Peripheral blood leukocytes (PBLs) were used for DNA isolation. Ten milliliters of blood were collected and centrifuged at 1,100 x g for 30 min at room temperature. The buffy coat was collected and subjected to osmotic hemolysis using cold water and 4.5% NaCl. PBLs were recovered by centrifugation at 600 x g for 10 min and washed twice in PBS. Genomic DNA was extracted using a DNeasy Tissue Kit (Qiagen), according to the manufacturer’s instructions.

### Serological study

The serological status of animals for SRLV infection was determined using the commercially available ID Screen MVV/CAEV Indirect Screening test (IDVet, France) according to the manufacturer’s recommendations. Additionally, sera were tested by ELISA based on Gag and Env multi-epitope recombinant antigens [18]. These antigens included either the Gag domain, containing nearly the whole matrix (MA) and capsid (CA) proteins fused to the SU1 and SU5 antigenic sites of the surface glycoprotein (SU)–(SU1/Gag/SU5 antigen), or the SU1 and SU5 antigenic sites only (without the Gag domain)–(SU1/SU5 antigen). Both antigens were developed on the basis of subtypes A1, A13, B1 and B2 of SRLV, which had been confirmed to circulate in small ruminants in Poland [18].

### PCR technique

For initial screening of provirus-positive sheep and goats, nested PCR amplification of the 625 bp fragment of the *gag* gene was performed. GAGf1 and P15 primers were used in the first round of the amplification [18], while CAGAG5 and CAGAG3 primers were employed in the second round [20]. For molecular characterization of proviral DNA, the V1/V2 (394 bp) and V4/V5 (608 bp) fragments of the *env* gene and a 990 bp fragment of the *gag* gene encoding MA/CA proteins, were amplified by nested PCR. Ptat and Penv primers were used in the first round of PCR for amplification of both the V1/V2 and V4/V5 fragments [18]. For the second round, A51/B31 and 567/564 primers were used for the amplification of the V1/V2 and V4/V5 fragments, respectively [14, 21]. For amplification of the *gag* gene fragment containing nearly the complete MA and the entire CA coding sequences, the following primer pairs were used: GAGf1 and P15 in the first round, and MA3f and NC3r in the second round [18]. If the amplification of the 990 bp *gag* gene fragment had failed, the 625 bp fragment corresponding to the CA coding sequence was amplified as described above. PCR products were analyzed by electrophoresis on 2% agarose gel containing ethidium bromide (1μg/ml) in 1xTAE buffer.

### DNA sequencing and sequence analysis

The amplicons of selected DNA samples were purified from agarose gels using the NucleoSpin Extract II kit (Marcherey-Nagel) and were cloned into the linear pSTBlue-1 AccerTor (Novagen), according to the manufacturer’s protocol. Five to seven clones representing each amplified fragment of each DNA sample were sequenced on a 3730x1 DNA Analyzer (Applied Biosystems) using a Big Dye Terminator v3.1 Cycle Sequencing Kit. The cloned sequences were aligned to obtain consensus sequences for each fragment of each animal (DNA sample) using the Geneious alignment module within Geneious Pro 5.3 software (Biomatters Ltd). Manual rearrangements of the alignments, including gap exclusion and length adjustment, were carried out to achieve optimal results. Consensus sequences were aligned to each other and with reference sequences. Phylogeny construction was performed using the Geneious tree-builder tool, and phylogenetic trees were constructed using Mr Bayes method with GTR substitution model. Pairwise genetic distances were calculated with the MEGA 6 software application [22] according to the p-distance substitution model with default settings, except for ignoring all the sites with gaps. All novel sequences representing SRLV isolates reported in this study are available under GenBank accession numbers KY864978–KY865033.

### Analysis of recombination

The Recombination Detection Program, version 3.44 (RDP3) was used to detect possible recombination events [23]. RDP3 provides access to seven primary exploratory recombination signal detection methods. These are the original RDP method, GENECONV, BOOTSCAN/RECSCAN, MAXCHI, Chimaera, 3SEQ and SISCAN. RDP3 is software that applies a number of recombination detection and analysis algorithms. The main strength of RDP3 is that it simultaneously uses a range of different recombination detection methods to both detect and characterize the recombination events that are evident within a sequence alignment without any prior user indication of a non-recombinant set of reference sequences.

## Results

### Serology and detection of provirus

Out of 59 serum samples, 47 (11 sheep and 36 goats) were positive in commercial ELISA while 23, 48, 46 and 41 were positive by ELISA based on SU1/Gag/SU5antigens representing subtypes B2, B1, A13 and A1, respectively. Overall, 56 serum samples (19 sheep and 37 goats) reacted with at least one recombinant antigen. When serum samples were tested by ELISA with SU1/SU5 antigens only, 11, 10, 13 and 8 were positive with antigens representing subtype B2, B1, A13 and A1, respectively. No positive response was detected in 38 (16 sheep and 22 goats) samples. Sera from two sheep and nine goats reacted to SU1/SU5 antigens derived from both A and B SRLV groups, suggesting co-infection with highly divergent viruses. For further study we selected 24 animals (10 sheep and 14 goats) with serological patterns suggesting infection with a new subtypes or co-infection with different viruses. Table 1 shows a summary of serological and PCR test results obtained from the 24 examined animals.

**Table 1:** Origins and characteristics of the Polish SRLV strains selected for genetic characterization.

Species	Flock origins	Animal No.	Commercial ELISA	PCR	Antigens							
					SU1/GAG/SU5				SU1/SU5			
					B1	B2	A1	A13	B1	B2	A1	A13
**Sheep**	1	**11**	+	+	+	+	-	-	-	-	-	-
		**2437**	+	+	+	+	-	-	+	+	-	-
		**4106**	+	+	+	+	+	+	-	-	-	-
		**4084**	+	+	+	+	-	+	-	-	-	-
	2	**2**	+	+	+	-	+	+	-	+	-	+
		**5**	-	+	-	+	+	-	-	-	-	-
		**6**	+	+	-	-	+	+	-	-	-	+
		**10**	+	+	-	-	+	-	-	-	-	-
		**13**	-	+	+	+	+	+	-	+	-	+
		**15**	-	+	-	+	+	-	-	-	-	-
**Goat**	3	**2461**	+	+	+	+	+	+	+	+	-	-
		**2462**	+	+	+	+	+	+	+	+	+	-
		**2466**	+	-	+	+	+	+	+	+	+	+
	4	**2991**	+	+	+	+	+	+	+	+	+	-
		**2993**	+	+	+	+	+	+	-	-	-	-
	5	**1202**	+	+	+	+	+	+	+	+	+	-
		**1203**	+	+	+	+	+	+	+	-	+	+
		**3085**	+	+	-	-	-	+	-	-	-	-
		**1561**	+	+	-	+	+	+	-	-	-	-
		**8370**	+	+	-	+	+	+	-	-	-	-
		**5616**	+	+	-	+	+	+	+	-	+	+
		**0042**	+	+	+	+	+	+	-	-	+	-
		**8344**	+	+	-	+	+	+	-	-	+	-
		**5675**	+	+	-	+	+	+	-	-	-	-

### Phylogenetic analysis of SRLV sequences

**Fig 1:**
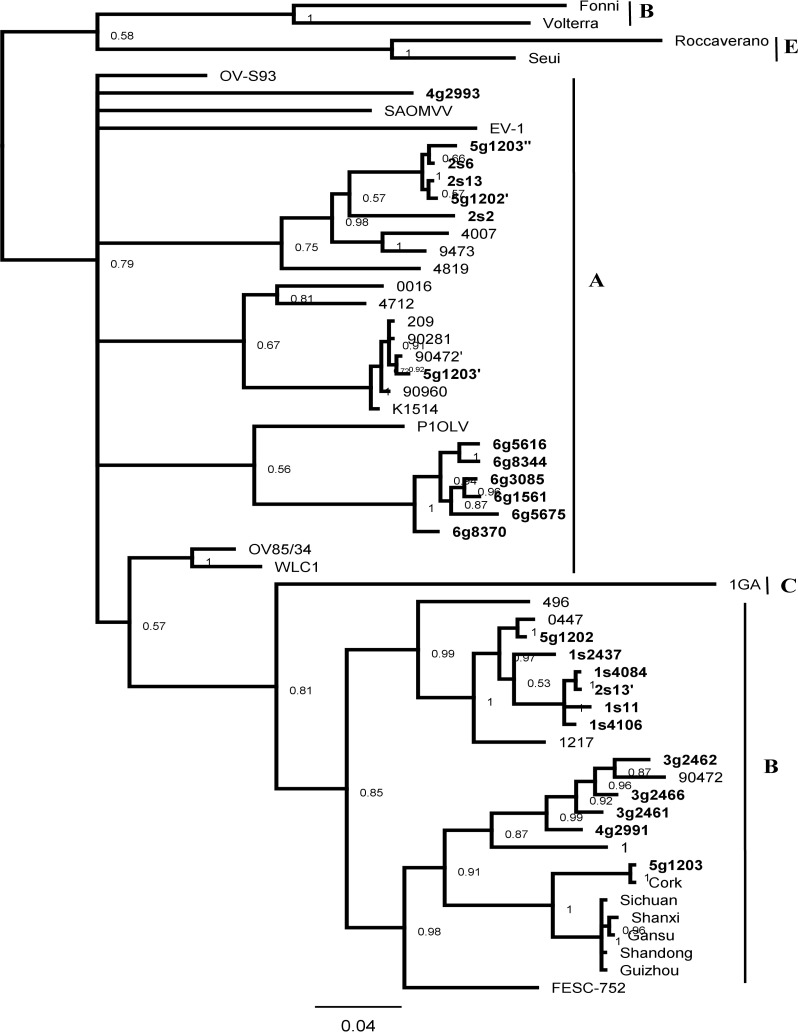
Bayesian phylogenetic tree based on V1/V2 SU fragment. Sequences from this study are shown in bold and their names are preceded by the flock origin No. and the animal species (s–sheep; g–goat). Sequences of co-infection variant strains are indicated by a (‘) and (“).

Amplification of the V1/V2 fragment gave specific products of the correct size in 20 out of 24 selected DNA samples (83.3%), including 7 sheep and 13 goats. We failed to amplify sheep samples s5, s10 and s15 from flock 2 and goat sample g0042 from flock 6. From each sample 5 to 7 clones were sequenced and analyzed to generate consensus sequences. In order to analyze the degree of genetic variability, consensus sequences were aligned with reference sequences from GenBank representing the genotypes described to date (groups A–E). We aimed to include as many geographically diverse strains as possible, however, we included only sequences of length matching data generated in our laboratory. An unrooted phylogenetic tree was constructed using the Mr Bayes method as shown in Fig 1.

The sequences derived from goats were widely distributed in the tree, clustering in subtype B1, B2, A12 or A1. Additionally, one and six sequences formed two new clusters within group A, which could be tentatively defined as A16 (strain g2993) and A17 (strains g8370, g5616, g8344, g5675, g3085 and g1561), respectively. The nucleotide divergence between isolates classified within these two new clusters ranged from 22.0% to 24.0% and the mean divergence between new subtypes and subtypes A1, A2, A12 and A13 varied from 19.6% to 24.1% for subtype A16 and from 17.4% to 22.4% for subtype A17. Additionally, the existence of new subtypes was supported by high bootstrap value (0.79). Thus, we propose to distinguish A16 and A17 as new subtypes within group A. Co-infections with highly divergent SRLV subtypes were found in two goats from flock 5 (subtypes A12/B2 in animal g1202 and subtypes A1/B1/A12 in animal g1203). Isolates derived from sheep were segregated into B2 and A12 subtypes and all isolates from the same flock clustered together within the same branch. Co-infection with subtypes A12 and B2 was found in one animal, sheep s13 from flock 2. In this study, the most frequently detected subtypes were A17 and B1, identified in 6 and 5 goats respectively and subtype B2, found in 5 out of 7 sheep.

**Fig 2:**
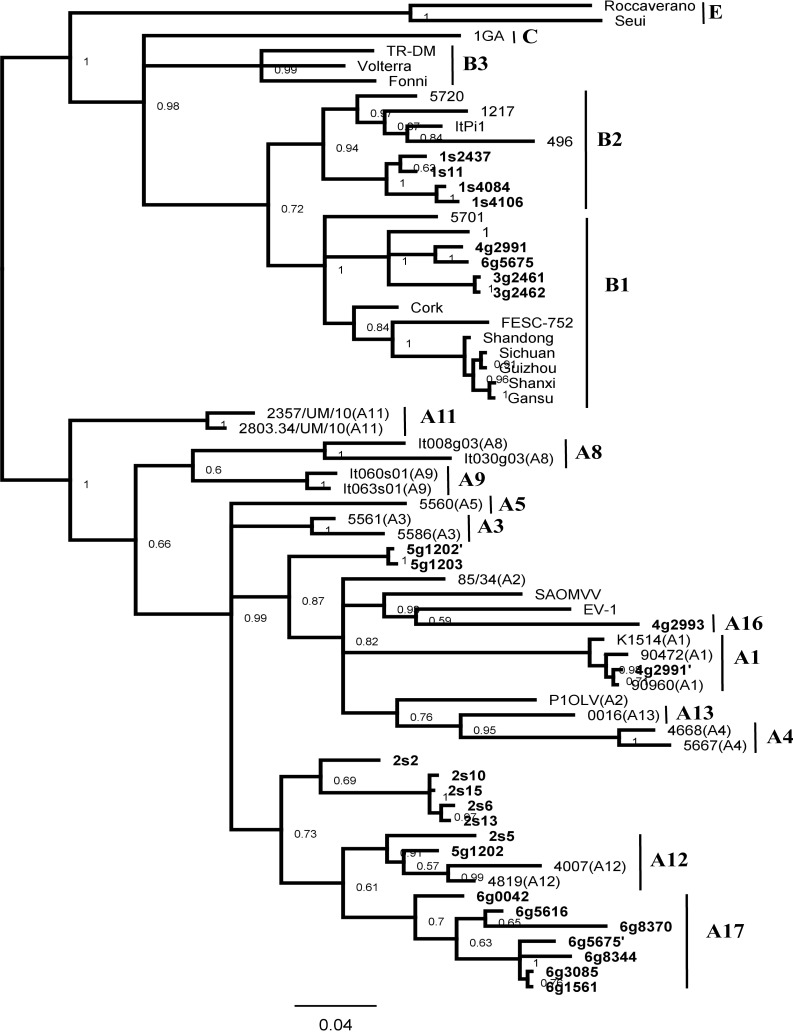
Bayesian phylogenetic tree based on CA fragment. Sequences from this study are shown in bold and their names are preceded by the flock origin No. and the animal species (s–sheep; g–goat). Sequences of co-infection variant strains are indicated by a (‘) and (“).

In order to confirm the genetic assessment of the analyzed lentiviruses, a 990 bp *gag* fragment covering the near-complete MA/CA coding region was amplified and then cloned for MA/CA sequence determination. We failed to amplify goat sample g2466 from flock 3. In case of sheep samples s11, s2437, s4106 and s4084 amplification of the 990 bp fragment was not possible, however, we successfully amplified a full-length fragment encoding the capsid protein. Phylogenetic analysis was first performed on the CA region (467 bp), for which sequences representing most SRLV subtypes were available. We excluded the sequences from genotypes D, A7, A6, A10 and A15 since sequences of these genotypes corresponding to the *gag* and *env* fragments are not available. Additionally, subtype A14 has not been included in the analysis since available sequences [24] represent a fragment shorter than 467 bp. The phylogenetic tree (Fig 2) indicated that the analyzed sequences belonged to subtypes B2, B1, A1 and A12 as well as to the newly identified subtypes A16 and A17. Similarly to the phylogenetic assignment inferred by the *env* sequences, *gag* sequences from goat g2993 formed the new subtype A16, while sequences from goats g0042, g5616, g8344, g8370, g3085, g5675 and g1561 formed subtype A17. The percentage of divergence between sequences belonging to the A16 subtype and those belonging to A17 subtype ranged from 15.5% to 16.9%, while the mean diversity between these subtypes and other subtypes of group A (A1-A5, A8-A9, A11-A13) varied from 15.7% to 18.7% and from 11.0% to 16.3% for A16 and A17, respectively (Table 2).

**Table 2:** Mean nucleotide distance in CA fragment of *gag* gene among (inter-genotype) genotypes A and B of SRLV.

	B1	B2	B3	A1	A2	A3	A4	A5	A8	A9	A11	A12	A13	A16
B1	-	-	-	-	-	-	-	-	-	-	-	-	-	-
B2	12.1	-	-	-	-	-	-	-	-	-	-	-	-	-
B3	18.9	17.4	-	-	-	-	-	-	-		-	-	-	-
A1	25.4	23.2	22.6	-	-	-	-	-	-	-	-	-	-	-
A2	23.6	21.4	22.1	17.4	-	-	-	-	-	-	-	-	-	-
A3	21.6	20.7	20.6	15.7	15.0	-	-	-	-	-	-	-	-	-
A4	22.7	21.3	22.9	16.6	16.6	16.0	-	-	-	-	-	-	-	-
A5	21.9	20.7	22.2	15.0	14.9	12.2	14.1	-	-	-	-	-	-	-
A8	23.6	22.7	22.3	17.3	17.6	15.9	17.0	17.7	-	-	-	-	-	-
A9	22.9	21.4	20.8	16.5	17.1	13.5	15.6	14.2	14.8	-	-	-	-	-
A11	21.6	22.0	21.4	16.2	17.0	15.0	18.2	15.7	16.4	14.4	-	-	-	-
A12	22.3	22.0	22.0	17.4	15.0	12.0	16.6	13.1	16.5	16.0	14.5	-	-	-
A13	23.0	20.5	21.5	17.2	14.2	13.5	13.4	14.8	17.0	16.5	15.8	13.5	-	-
**A16**	**21.7**	**22.0**	**23.5**	**17.7**	**16.3**	**17.2**	**15.7**	**15.7**	**17.8**	**18.7**	**18.1**	**17.0**	**16.4**	**-**
**A17**	**22.0**	**20.9**	**21.1**	**15.1**	**14.6**	**11.0**	**15.0**	**12.5**	**16.3**	**12.8**	**15.7**	**12.5**	**15.0**	**16.3**

Sequences generated from goat g5675 were affiliated not only to subtype A17 but also to B1, which indicated co-infection in this animal. A similar status was seen in goat g2991, which was co-infected by viruses classified within A1 and B1 subtypes. Sequences of goats g1202 and g1203 formed separate clusters within group A, closely related to A3 subtypes, with mean nucleotide distance of 10.5%. Sequences of sheep s6, s13, s10, s15, and s2 also formed separate clusters within group A and were closely related to subtype A12. Since these sequences have not been clearly distinguished on the tree constructed based on V1/V2 sequences, their classification within new subtypes is not further supported. An additional argument against classification of these isolates within the new subtypes is that sequence differences were below the threshold of 15% set by Shah et al. [5] as a criterion to define new subtypes of SRLV.

**Fig 3:**
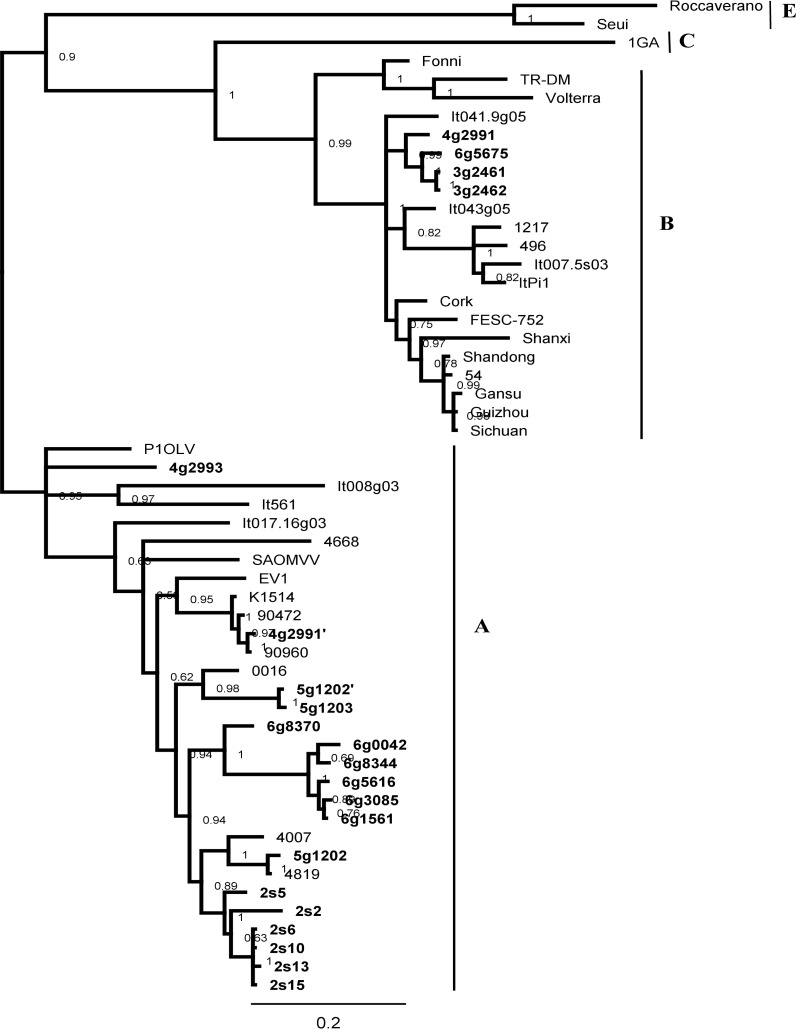
Bayesian phylogenetic tree based on MA fragment. Sequences from this study are shown in bold and their names are preceded by the flock origin No. and the animal species (s–sheep; g–goat). Sequences of co-infection variant strains are indicated by a (‘) and (“).

Phylogenetic analysis was also performed on the basis of the MA region. As shown in Fig 3, the overall tree topology was consistent with that constructed from V1/V2 and CA, supporting the existence of new subtypes A16 and A17. The mean nucleotide distance between strain g2993 from subtype A16 and strains representing subtype A17 was 19.1%. The mean distance between A16 and A17 subtypes and all other strains within group A were 16.1% and 19.6% respectively.

### Analysis of recombination

No evidence of recombination was found for the *gag* gene sequences based on RDP analysis. Analysis of *env* sequences (V4 and V5) showed some indications of recombination in samples originating from flocks 4, 5 and 6. However, these possible recombination events were not considered credible, since the program was not confident on the proposed recombination event, its location and sequence breakpoints, nor its contributing sequences.

### Antigenic characterization of Polish strains

**Fig 4:**
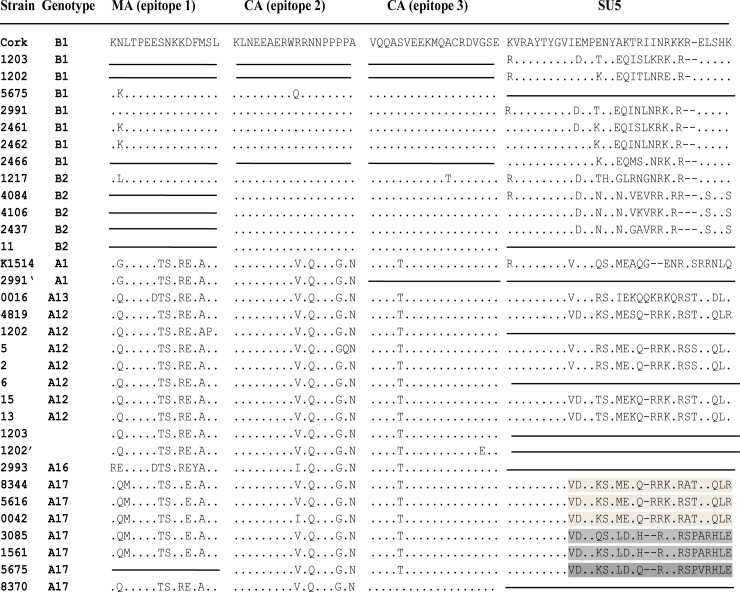
Sequence homology of Gag and SU immunodominant regions between Polish strains analyzed in this study with reference strains from genotypes A1(K1514), B1(Cork), B2(1217), A13(0016) and A12(4819). Deletions are indicated by a dash (-) and identical residues are indicated by dots (.). Line indicated that sequences are not available.

In order to analyze amino acid (aa) sequences of immunodominant regions of strains analyzed in this study, the deduced aa sequences of matrix, capsid and surface proteins were aligned with the respective aa sequences of reference parental strains and strains representative for subtypes previously found in Poland (Fig 4). The immunodominant epitopes 1 and 2 located in the matrix and capsid proteins, respectively, clearly showed group-specific variability while the immunodominant epitope 3, situated at the C-terminal end of the capsid protein, was almost identical in all strains. All strains belonging to group A had two aa deletions (GG) in the middle part of the CA protein and a seven aa insertion (QLYPNLE) which was typical for strains belonging to this group. Comparison of aa sequences in the SU5 epitope revealed perfectly conserved region located at the N-terminal part of the epitope (VRAYTYGVI) and a highly variable region located at the C-terminal part of the epitope. The variable region was conserved among the strains belonging to subtype B1 and subtype A12, showing type-specific variation. High sequence homology was also seen among strains s4084, s4106 and s2437, belonging to subtype B2. However, they differed significantly from the sequence of previously characterized B2 strain 1217 [18] used as a reference sequence in this study. Analysis of sequences of a new A17 subtype showed the existence of two clearly separated groups. The sequences of strains g8344, g5616 and g0042 (group I) showed almost 100% identity to each other whereas sequences of strains g3085, g1561 and g5675 (group II) varied by three aa substitutions. Both groups showed a common motif located at the N-terminal part of the epitope (KVRAYTYGVVDMPK/QSY) and different motifs at the C-part of the epitope specific for group I (METQ-RRKKRA/STELQLR) and group II (LDTH/Q—RRKRSPA/VRHLE).

Analysis of immunoreactivity of epitopes in the ELISA with SU1/Gag/SU5 proteins showed cross-reactive antibodies to group A and B-derived Gag proteins (Table 1). 20 out of 24 analyzed sera reacted to subtype B2 and A1, 19 reacted to subtype A13 while 14 showed the reactivity to B1-derived antigen. When serum samples were tested with ELISA based on SU1/SU5 proteins, only 8 sera were positive with antigens representing subtype B2, B1 and A1, while 6 reacted to subtype A13. No positive response to SU1/SU5 antigens representing subtypes B1, B2, A1 and A13 was detected in 11 serum samples. These sera which eluded serological detection were collected from animals infected with strains belonging to B2 (s4084, s4106 and s11) and A12 (s15, s5, s10) subtypes as well as to newly identified subtypes A16 (g2993) and A17 (g1561, g3085 and g5675). Sequence analysis of SU5 epitope of those strains confirmed that they were highly divergent from reference strains (K1514(A1), Cork(B1), 1217(B2) and 0016(A16) used to construct SU1/SU5 recombinant antigens.

## Discussion

Previous phylogenetic analyses of SRLV sequences found in Poland revealed the circulation of subtype A1 in both sheep and goats, subtypes B1 in goats and subtypes B2, A12 and A13 in sheep [18]. In this study, we presented a more detailed picture of SRLV strains circulating in sheep and goats from Poland. We showed that analyzed strains were highly heterogeneous and represented ovine strains belonging to subtypes A12 and B2 and caprine strains grouped in subtypes B1, B2, A1 and A12. We also revealed the existence of new subtypes circulating in goats. The sequence of strain g2993 from flock 4 and those of strains g0042, g5616, g8344, g5675, g3085, g8370 and g1561 originating from flock 6 were affiliated to group A, however, they were segregated into two clusters, distinct from known subtypes. The mean pairwise genetic distances of *gag* and *env* sequences of both clusters were above 15% nucleotide divergence when compared to all other subtypes within group A, which is a criterion required to distinguish a new subtype [5]. Additionally, the existence of two separated clusters was confirmed by high bootstrap values. Thus, we propose to designate these two clusters as new subtypes, A16 and A17.

Many studies have documented the circulation of SRLV in both sheep and goats as a result of cross-species infection [5, 25] and the infection of goats with group A viruses (MVV-related viruses) representing A1, and A3 to A14 [6]. It is generally accepted that common breeding of sheep and goats in traditional mixed flocks is the factor promoting transmission of SRLV [13, 25]. However, in our study we selected animals from single-species flocks, and so the presence of goats infected with A16 and A17 subtypes presents unequivocal evidence for the transmission of these viruses in the past. Infection with new subtypes may possibly lead to virus adaptation to a new host and promote changes of its biological and pathogenic properties [6]. It was shown that viruses representing the A4 subtype were generally attenuated in naturally infected goats, however, they caused histopathological changes in the mammary tissue [26]. It was remarkable that the A17 subtype was present in 7 out of 23 goats from flock 6, which could suggest broad dissemination of this subtype. Whether viruses belonging to the A16 and A17 subtypes exhibit new properties remains to be elucidated.

Mixed infections with lentiviruses belonging to different subtypes were documented in humans infected with HIV-1 [27] and cats infected with FIV [28]. Circulation of more than one SRLV genotype in goats was reported by Kuhar et al. [24] and in sheep and goats by several authors [7, 8, 18, 29]. In the present study in four flocks, co-infection with strains belonging to different subtypes within A and B groups was evidenced in 1 sheep and 4 goats originating from 4 flocks. One sheep (s13) from flock 2 and one goat (g1202) from flock 5 were co-infected with B2/A12 subtypes while another three goats (g2991, g1203 and g5675) originating from flocks 4, 5 and 6 were co-infected with the B1/A1, B1/A1/A12 and B1/A17 subtypes, respectively. Co-infection events in these animals were confirmed by both phylogenetic analysis and serological reactivity towards SU1/SU5 recombinant antigens derived from SRLV groups A and B.

In four animals, despite their serological reactivity to both group A and B-derived antigens suggested co-infection, phylogenetic analysis did not confirm the fact of co-infection. According to L’Homme et al. [8], the existence of co-infected animals and flocks harboring a range of different subtypes of SRLV can be at least partially explained by the introduction into a flock of infected animals from other sources. In fact, in our study co-infection of sheep with A12 and B2 subtypes probably had its source in the mixing of animals coming from flocks 1 and 2, which are parts of the station of the University of Life Sciences in Lublin. The presence of multiple subtypes in one animal always leads to speculation whether infection occurs sequentially, as a superinfection, or simultaneously, as a dual-infection event. We can hypothesize that goat g1203 was superinfected with subtype A12, since in our previous study performed in the same flock [18], we showed the circulation of A1 and B1 subtypes exclusively.

Co-infection with more than one lentivirus genotype offers an opportunity for viral recombination. Under natural conditions, recombination events between SRLV genetic groups A and B [7, 8, 29, 30] and between subtypes within the same group (B) in goats have been identified [31]. In the present study, the similarity in the plots of *gag* gene sequences did not reveal any evidence of recombination, but on the basis of *env* sequences (V4 and V5) we observed some indications of recombination in samples originating from flocks 4, 5 and 6. Although we cannot rule out that recombination events took place, the results of the present study did not provide sufficient evidence that recombination had occurred. The lack of recombination event found in this study can be possibly linked to the infection distribution found in the present study, where 80% of tested animals were infected with a single subtype only. In contrast, frequent recombination events have recently been reported in sheep and goats from Canada, where co-infection with more than one SRLV strain may not be uncommon [29].

An important drawback of SRLV serology is the limited capacity for detection of specific antibodies generated by different subtypes, especially newly emerging ones. In this study sera from sheep and goats infected with different SRLV subtypes, including newly identified A16 and A17 subtypes, were tested by ELISA with SU1/Gag/SU5 and SU1/SU5 antigens derived from A1, A13, A12, B1 and B2 subtypes. Immunological relatedness was found between Gag epitopes of subtypes used for the construction of antigens for ELISA and all subtypes tested in our study, what confirmed the cross-reacting nature of Gag antibodies [11]. Unexpectedly, almost all sera from animals infected with subtype A17 reacted with Gag antigen derived from the B2 subtype, despite the fact that these two subtypes showed a high degree of heterogeneity (20.9%). This may be explained by the fact that the spectrum of antibody reactivity to the Gag antigens is larger in goats than in sheep [32].

Many studies showed that the variability of the SU5 epitope in different SRLV subtypes may account for the type-specific immune response allowing strain-specific diagnosis [14, 33, 34]. As expected, the majority of tested sera reacted with the antigen corresponding to the SU5 aa sequences of homologous subtypes. However, some of these sera cross-reacted with SU5 antigen derived from different subtypes and this can be explained by a low avidity between an antibody and conserved amino acid domain shared by the SU5 sequences of different subtypes [14]. Reactivity of these sera to antigens derived from different subtypes can be additionally explained by co-infection with strains belonging to the different subtypes.

In this study, we found 11 sera that failed to react with the SU1/SU5 antigens, most probably because of the large antigenic divergence of SU5 antigens between the infecting viral strains and those used in ELISA. The lack of ability of antibodies to bind to antigens indicate that sera bind not only to the conserved region of SU5 but rather to both constant and variable regions of the SU5 epitopes and that aa sequence of variable region presumably determines the avidity of the reaction[14].We can suppose that the SU5antigens, representing four SRLV subtypes, used in this study, may not cover the whole SRLV antigenic spectrum and failed to detect emerging viruses.

In summary, the results of this study show that sheep and goats in Poland can be infected by a range of SRLV subtypes and some animals can be co-infected with viruses belonging to different subtypes. Phylogenetic analysis revealed the existence of new subtypes, divergent from previously known subtypes of group A. In order to gain a better understanding of SRLV global genetic diversity, it is essential to include as many geographically diverse isolates as possible. Because in the case of new subtype A16 only one sequence was detected, additionally study will be performed to definitely confirm the occurrence of this subtype in Polish flocks. We expect further clarity to sub-clades and perhaps even the discovery of more novel genotypes as further isolates will be characterized. This highlights one of the limiting factors in the study of SRLV: essentially the lack of complete sequence data which as can be seen here is not only important for elucidating phylogenetic relationships but also in highlighting potential effects on serological detection. Moreover, further isolation and characterization of viruses belonging to the new subtypes may shed a light on their biological and virological properties.
